# Extrapolating neurogenesis of mesenchymal stem/stromal cells on electroactive and electroconductive scaffolds to dental and oral-derived stem cells

**DOI:** 10.1038/s41368-022-00164-6

**Published:** 2022-02-24

**Authors:** Boon Chin Heng, Yunyang Bai, Xiaochan Li, Xuehui Zhang, Xuliang Deng

**Affiliations:** 1 Central Laboratory, Peking University School and Hospital of Stomatology, Beijing, China; 2 School of Medical and Life Sciences, Sunway University, Subang Jaya, Selangor Darul Ehsan Malaysia; 3 Department of Geriatric Dentistry, Peking University School and Hospital of Stomatology, Beijing, China; 4 Department of Dental Materials & Dental Medical Devices Testing Center, Peking University School and Hospital of Stomatology, Beijing, China; 5 National Engineering Laboratory for Digital and Material Technology of Stomatology, NMPA Key Laboratory for Dental Materials, Beijing Laboratory of Biomedical Materials & Beijing Key Laboratory of Digital Stomatology, Peking University School and Hospital of Stomatology, Beijing, China

**Keywords:** Stem cells, Stem-cell research, Dentistry

## Abstract

The high neurogenic potential of dental and oral-derived stem cells due to their embryonic neural crest origin, coupled with their ready accessibility and easy isolation from clinical waste, make these ideal cell sources for neuroregeneration therapy. Nevertheless, these cells also have high propensity to differentiate into the osteo-odontogenic lineage. One strategy to enhance neurogenesis of these cells may be to recapitulate the natural physiological electrical microenvironment of neural tissues via electroactive or electroconductive tissue engineering scaffolds. Nevertheless, to date, there had been hardly any such studies on these cells. Most relevant scientific information comes from neurogenesis of other mesenchymal stem/stromal cell lineages (particularly bone marrow and adipose tissue) cultured on electroactive and electroconductive scaffolds, which will therefore be the focus of this review. Although there are larger number of similar studies on neural cell lines (i.e. PC12), neural stem/progenitor cells, and pluripotent stem cells, the scientific data from such studies are much less relevant and less translatable to dental and oral-derived stem cells, which are of the mesenchymal lineage. Much extrapolation work is needed to validate that electroactive and electroconductive scaffolds can indeed promote neurogenesis of dental and oral-derived stem cells, which would thus facilitate clinical applications in neuroregeneration therapy.

## Introduction

To date, numerous adult mesenchymal stem/stromal cell lineages have been identified and extracted from the oral cavity. These include dental pulp stem cells (DPSCs), dental follicle stem cells (DFSCs), stem cells from human exfoliated deciduous teeth (SHED), periodontal ligament stem cells (PDLSCs), stem cells from apical papilla (SCAP) and gingival mesenchymal stem/stromal cells (GMSCs).^1,2^ In recent years, these adult stem cells have demonstrated much promise for tissue engineering and regenerative medicine applications, due to their extensive multi-lineage differentiation potential, as well as their ready accessibility and ease of isolation from clinical waste produced during routine dental treatment.^1,2^ In particular, the high neurogenic capacity of these cells due to their embryonic neural crest origin,^3^ make them especially attractive for neuroregeneration therapy of traumatic injuries to the brain, spinal cord and peripheral nervous system, as well as cerebrovascular and neurodegenerative diseases.^4^ Nevertheless, neuroregeneration within the dental pulp of diseased/damaged tooth has largely been overlooked, which will be discussed in the next section.

It is important to note that dental and oral-derived stem cells also possess a high propensity to differentiate into the osteo-odontogenic lineage, which could in turn compromise their neurogenic differentiation capacity. Hence, previous studies have investigated various strategies to enhance the neural differentiation of these adult cells, involving supplementation of various growth factors and small molecules within the culture milieu,^5^ recombinant gene expression,^6^ utilization of various novel scaffold materials,^7,8^ together with the application of physical stimuli such as ultrasound.^8^

**Fig. 1:**
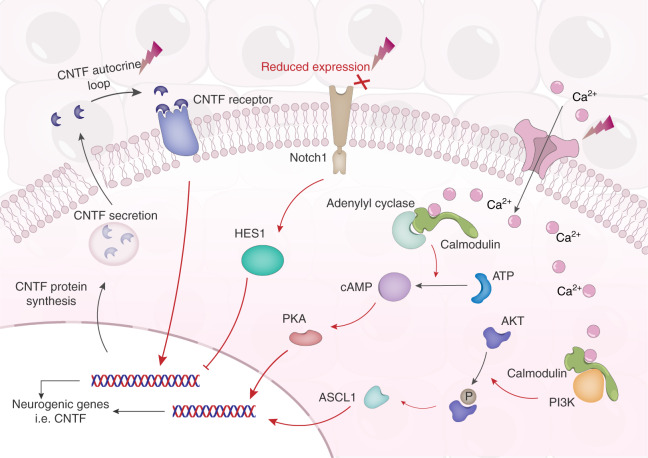
Pro-neurogenic signaling pathways activated by electrical stimuli, via voltage-gated calcium channels or cell surface receptors such as Notch1 and the CNTF receptor. Adapted from Heng et al.^19^. The red pointed arrows denote enhancement, while the red blunted arrows denote inhibition. The black pointed arrows denote chemical transformation or movement of molecules

To date, various biophysical properties of scaffold materials have been reported to influence the neurogenic differentiation of stem cells, including mechanical properties such as stiffness,^9^ elasticity,^10^ surface roughness^11^ and topography,^12^ as well as bioelectical properties such as piezoelectricity,^13^ static electrical charge^14^ and conductivity.^15^ The major difference in the way mechanical properties influence neurogenesis of stem cells, as compared to bioelectrical properties, lies with the signaling transduction pathways involved. While neurogenesis induced by mechanical stimuli involves suppression of the canonical mechanotransduction signaling axis composed of focal adhesions, cytoskeletal stress fibers, and nuclear translocation of YAP/TAZ transcriptional co-activators;^16,17^ neurogenesis induced by electrical stimuli involves activation of various pro-neurogenic signaling pathways triggered by voltage-gated ion channels and cell surface receptors (section “Signaling pathways implicated in electrical stimulation of neurogenesis”, Fig. 1).

Because, neural tissues are well known to be electroactive and electrogenerative, being capable of generating action potential; recapitulating the natural physiological electrical microenvironment of neural tissues via electroactive or electroconductive scaffolds would be a biomimetic approach to enhance the neurogenesis of dental and oral-derived stem cells. It is important to distinguish between electroactive and electroconductive scaffolds. Electroactive scaffolds refer to scaffolds with piezolelectric properties or static electrical charge, whereas electroconductive scaffolds refer to scaffolds without these two aforementioned properties, but with the capacity to conduct electricity (Table 1).

At this juncture, it would be appropriate to compare the various advantages and disadvantages of utilizing dental and oral-derived MSCs versus other MSC sources for neuroregenerative therapy. One major advantage is the much high proliferative potential of some dental/oral-derived MSC lineages such as SHED, DFSCs, and SCAPs, as compared to MSCs derived from bone marrow.^18,19^ Utilizing BrDU immunoassay and cell counts, Kunimatsu et al.^18^ found that SHED was much more highly proliferative compared to DPSCs and bone marrow-derived MSCs. Similarly, Tamaki et al.^19^ reported that both DFSCs and SCAPs possesed much higher proliferative potential than bone marrow-derived MSCs, based on cell counts and Propidium Iodide staining analysis of cell cycle via flow cytometry. Another advantage could be the higher neurogenic potential of dental/oral-derived MSC lineages, as compared to other sources of MSCs. Li et al.^20^ reported that GMSCs and DPSCs exhibited functional neural-like electrophysiological properties after 3D neurosphere culture, with K+ and Na+ currents being detected by patch clamp. By contrast, such properties were not displayed by bone marrow-derived MSCs that were subjected to the same 3D neural induction culture protocol.^20^ Foudah et al.^21^ reported that undifferentiated DPSCs and PDLSCs expressed much higher levels of the key neural stem cell marker nestin, as compared to undifferentiated MSC obtained from adipose tissue and skin. Kumar et al.^22^ showed that treatment of the pre-neuroblastic cell line IMR-32 with the secretome of DPSCs, DFSCs, and SCAPs, resulted in a more significant enhancement of neurogenic differentiation, as compared to treatment with the secretome of bone-marrow-derived MSCs. Nevertheless, the major disadvantage of dental/oral-derived MSCs are their less ready availability as an autologous source, compared to MSCs derived from bone marrow and adipose tissue; as these are the by-product of dental treatment, and it would be less practical to extract these from healthy dental/oral tissues without substantial donor site morbidity.

To date, there had been very few studies on the pro-neurogenic effects of electroactive/electroconductive scaffolds on dental and oral-derived stem cells. Most of the relevant scientific information comes from the study of other mesenchymal stem/stromal cell lineages (particularly bone marrow and adipose tissue) cultured on electroactive and electroconductive scaffolds, which will therefore be the focus of this review (Tables 1–4). Although there had been much larger number of similar studies on neural cell lines (i.e. PC12), neural stem/progenitor cells and pluripotent stem cells, the scientific data from such studies are much less relevant and less translatable to dental and oral-derived stem cells, which are of the mesenchymal lineage.

**Table 1:** Direct electrical stimulation on scaffolds for enhancing neurogenic differentiation

Electroconductive scaffold material	Electric stimuli	Mesenchymal stem/stromal cell source	Species	Neural markers assessed	ref.
Polyaniline (PANI)	Intermittent D/C for 10 min at 24 h intervals	Not specified	Human	βIII-tubulin, Nestin	^26^
Electroactuated gold nanoparticles	(1) Steady-state D/C electric field of 100 mV·cm^−1^ for a duration of 15 min every day, (2) Intermittent square pulses (10, 1, and 0.1 Hz), with a duty cycle of 10% and field strength of 100 mV/cm for 15 min every day	Not specified	Human	βIII-tubulin, Nestin, MAP2, NEFL, GFAP	^27^
Graphene	Cyclic voltammetry 0.5 V, 0.3 V, and 0.1 V, (1 Hz, 3 Hz, and 5 Hz)	Bone Marrow	Human	MAP2, βIII-tubulin	^28^
Polycaprolactone (PCL) nanofibre	4 Hz positive monophasic pulse wave with a 2.5 ms pulse duration and an amplitude of 1.1 V	Adipose	Mouse	Nf-L, Nf-M, Nf-H, SYP), NCAM, GAD, NeuN, βIII-tubulin, and MAP2	^29^
Polymethylmethacrylate (PMMA)	D/C ~(8 ± 0.06) mV·mm^−1^, continuously for 9 days at an exposure of 20 h per day, with a short intermittent resting phase of 4 h for each 24 h	Wharton jelly	Human	SOX2, Nestin, βIII-tubulin	^30^
Graphene cross-linked collagen cryogel	1 V D/C for 5 min (0.20 V·mm^−1^)	Bone marrow	Rat	Nestin, MAP2, βIII-tubulin, NeuN	^31^
Electrospun carbon nanotube/poly(*p*-dioxanone) (PPDO) composite nanofibers	50 mV·mm^−1^ for 1 h per day, from days 4 to 14 of culture	Adipose tissue	Human	S100b, MBP, GFAP, SOX10, NGFR, NCAM1, FABP7, MP7, MAG	^54^
Aligned electrospun polypyrrole/ polylactide composite nanofibers	100 mV·mm^−1^ for 30 min per day for 5 days	Umbilical cord	Human	Nestin, NF-L	^55^
Black-phosphorus incorporated gelatin methacryloyl hydrogel	100 mV·cm^−1^ for 20 min per day for 1 week	Bone marrow	Rat	Nestin, Tuj1, GFAP, MAP2,	^56^

**Table 2:** Electroactive scaffolds for enhancing neurogenic differentiation of mesenchymal stem/stromal cells

Electroactive scaffold material	Mesenchymal stem/stromal cell source	Species	Neural markers assessed	ref.
Poly (3,4-ethylenedioxythiophene) (PEDOT)-reduced graphene oxide (rGO) hybrid microfiber (80 μm in diameter)	Bone marrow	Rat	Tuj1, GFAP	^32^
CoFe_2_O_4_ (CFO)-polyvinylidene difluoride (PVDF) nanocomposite	Adipose tissue	Human	Nestin, βIII-tubulin, NSE, β-Sarcomeric actin	^33^
Agarose–alginate–chitosan–oligoaniline composite hydrogel	Olfactory mucosa	Human	MAP2, TH, DAT, Nurr1, Wnt1, Pitx3	^34^
Polyvinylidene difluoride (PVDF)-BaTiO_3_-multi-walled carbon nanotube (MWNT) nanocomposite	Bone marrow	Human	Nestin, βIII-tubulin, GFAP, MAP2	^35^

**Table 3:** Graphene-based electroconductive scaffolds for enhancing neurogenic differentiation of mesenchymal stem/stromal cells

Electroconductive scaffold material		Mesenchymal stem/stromal cell source	Species	Neural markers assessed	ref.
Graphene monolayer		Adipose	Human	Nestin, Tuj1, NeuN, GFAP	^43^
Graphene monolayer		Bone marrow	Human	Nestin, Tuj1, NF-L	^44^
Fluorinated graphene		Bone marrow	Human	Nestin, MAP2 Tuj1,	^45^
Graphene oxide		Adipose	Human	Tuj1	^46^
Graphene oxide		Adipose	Human	BDNF, GDNF, NGF	^47^
Graphene and natural polymer composite scaffolds	Collagen-coated graphene foam	Bone marrow	Mouse	β-III tubulin, TH, NeuN	^48^
	Reduced graphene oxide – porcine acellular dermal matrix	Bone marrow	Rat	Nestin, GFAP, MAP2	^49^
Graphene and synthetic polymer composite scaffolds	Electrospun polycaprolactone-graphene nanocomposite	Bone marrow	Human	β-III tubulin, TH, MAP-2	^50^
	Graphene- polycaprolactone -gelatin nanofiber	Bone marrow	Rat	O4, O1, MOG	^51^
	Graphene -polylactide-*co*-glycolide	Bone marrow	Human	Nestin, GFAP, TUJ1, MAP2	^52^
	Polyethylenimine (PEI) grafted graphene oxide	Bone marrow	Rat	β-III tubulin	^53^
	Graphene oxide–poly (acrylic acid) nanocomposite hydrogel	Bone marrow	Rat	GFAP	^54^
Graphene-augmented ceramic composite scaffold	Graphene-augmented inorganic metal oxide ceramic	Adipose	Human	Nestin, GFAP, Tuj1	^55^

**Table 4:** Carbon nanotube (CNT)-based electroconductive scaffolds for enhancing neurogenic differentiation of mesenchymal stem/stromal cells

Electroconductive scaffold material		Mesenchymal stem/stromal source	Species	Neural markers assessed	ref.
Carbon nanotube	Multiwall	Bone marrow	Human	GFAP, MAP2, NFL, NFM, NFH, β-III tubulin, Nestin,Synaptophysin	^56^
	Single-wall	Bone marrow	Human	Nestin, GFAP, MAP2, Tuj1	^57^
	Multiwall	Bone marrow	Human	β-III tubulin, NSE, GAP43, NFL, MAP1b, MAP2	^58^
	Multiwall	Bone marrow	Human	NF-L, GFAP	^59^
Carbon nanotube composite	Poly-lactic acid with alginate-gelatin and multiwall carbon nanotube coating	Wharton jelly	Human	Nestin, MAP2, NSE	^60^
	Single-wall carbon nanotube-pyrimethamine	Adipose	Human	NSE, NFM	^61^
	Multiwall carbon nanotube-sericin	Bone marrow	Mouse	Tuj1	^62^
	Poly (lactic-*co*-glycolic acid) microspheres containing single-wall carbon nanotubes dispersed in hyaluronic acid-poloxamer-ethoxy-silane-capped poloxamer and cross-linked alginate hydrogel	Adipose	Rat	Nestin, SOX II, βIII-tubulin, Synaptophysin	^63^

## Physiological role of tooth innervation

First of all, we need to understand the physiological role of peripheral nerves in tooth, and the need for neuroregeneration within the dental pulp of damaged/diseased tooth. The dental pulp is a highly innervated tissue.^23^ Besides its sensory function, the neurons of the dental pulp also play other important roles in tooth function and homeostasis.^24–26^ In particular, dental pulp neurons are closely associated with blood vessels,^23^ thus enabling them to control blood flow^25^ and infiltration of immune cells to the teeth,^26^ thereby mediating immunological defense against oral pathogens and healing upon tooth disease or injury.^24,26^ Despite their important functions, neural regeneration of the dental pulp does not occur spontaneously upon tooth injury or disease, and standard root canal treatment often results in the complete obliteration of neurological elements of the tooth, with consequent post-treatment complications. Hence, besides angiogenesis and odontogenesis, neurogenesis is also another important aspect of dental pulp regeneration, which has largely been overlooked (Tables 1–4).

A previous study demonstrated that the success of dentin-pulp regeneration is dependent on the sprouting of nerve fibers within the dental pulp cavity.^27^ This nerve sprouting enhances tooth healing by regulating the vascular permeability and recruitment of immune cells at the injury site.^28^ In pulpectomy experiments, a close relationship between nerve fibers and dentine bridge formation was observed.^29^ Indeed, a tooth injury experimental model showed more necrosis of dental pulp in tooth without nerves, as compared to tooth with nerves.^30^ Previous studies have demonstrated that dental stem cells can generate nerves upon transplantation into the pulp cavity of tooth,^31^ as well as secrete neurotrophic factors.^32^ Nevertheless, neuroregeneration within the dental pulp of diseased and damaged tooth has largely been overlooked, with most studies on dental pulp regeneration focusing only on angiogeneis or odontogenesis. There is a dire need for more research studies in this area (Tables 1–4).

## Signaling pathways implicated in electrical stimulation of neurogenesis

To date, a number of different molecular signaling pathways have been implicated in the electrical stimulation of neurogenesis (Fig. 1), as described in detail by our review article.^33^ Most probably, there are subtle differences in electrical stimulation of neurogenesis by the diverse variety of electroactive and electroconductive scaffolds composed of different materials. But as yet, there have been no rigorous and systematic comparisons of pro-neurogenic signaling pathways activated by electrical stimuli originating from the various different scaffold types. If major differences exist, these would most likely be due to varying magnitude of electrical stimuli from the different scaffold types. For example, the amplitude of alternating or direct electric current flowing through an electroconductive scaffold would be much higher compared to static electrical charge, or piezoelectricity generated by the migration and spreading of cells. Otherwise, it is expected that all electrical stimuli would be transduced into biological signals by voltage-gated ion channels^34,35^ or cell surface receptors (i.e. Notch1^36^ and CNTF receptor^37^) via a number of pro-neurogenic signaling pathways (Fig. 1). These include the cAMP-PKA signaling cascade,^34,38^ the PI3k-Akt signaling cascade,^39^ the Notch signaling cascade,^36^ and autocrine CNTF signaling,^37^ which have been described in detail in our review article.^33^ Based on the scientific literature,^39–41^ it would appear that both transmembrane and soluble isoforms of adenylyl cyclase play key roles in neuronal function and neurogenic differentiation via the cAMP-PKA signaling axis. Nevertheless, it is still unclear which of these isoforms play a more prominent role in electrical stimuli-induced neurogenic differentiation, which would need to be investigated by further research studies.

## Neural lineages derived from mesenchymal stem cells exhibit functional electrophysiological properties

An important prerequisite for utilizing MSCs for neuroregenerative therapy would be validate that these cells can indeed give rise to neural lineages with functional electrophysiological properties that are equivalent or similar to that of natural neural tissues, in addition to expressing key neural markers at the mRNA and protein level. Otherwise, without the capacity to exhibit functional electrophysiology, MSCs would be incapable of facilitating the regeneration of neural defects in vivo, despite the expression of appropriate markers and neural phenotype. Fortunately, to date, numerous studies have demonstrated that both dental and non-dental sources of MSCs are capable of developing functional neural-like electrophysiology upon neurogenic differentiation. Ullah et al.^42^ compared the neurogenesis of different MSC lineages isolated from dental tissues (follicle, papilla, and pulp) on the basis of electrophysiology and synaptic marker expression, and found that dental pulp-derived MSCs exhibited the best neurogenic differentiation potential among the three lineages, in terms of higher Na^+^ and K^+^ currents measured by patch clamp, as well as higher expression of synaptic markers. However, in the study of Li et al.^20^, it was reported that gingival-derived MSCs (GMSCs) had higher neurogenic potential compared to MSCs that were derived from either dental pulp (DPSCs), apical papilla (SCAPs), or bone marrow (BMSCs), as demonstrated by higher expression of neural markers, as well as superior electrophysiological properties from patch-clamp experiments. Only 3D neurosphere culture could yield neural-lineage cells that displayed functional action potential from GMSCs and DPSCs, but not BMSCs or SCAPs, with 21.2% of GMSCs-derived neuronal cells displaying action potential, versus only 8.3% of DPSCs-derived neuronal cells.^20^ In another study by Zhang et al.^43^, it was demonstrated that GMSCs seeded on 3D bio-printed scaffold could promote rat facial peripheral nerve regeneration, with the implanted GMSCs-derived grafts displaying similar compound muscle action potential as the autograft transplantation group upon nerve stimulation at 12 weeks post-transplantation. With regards to non-oral and non-dental sources of MSCs, the study of Subbarao et al.^44^ showed that porcine endometrium-derived MSCs displayed active K^+^ and Na^+^ currents after neurogenic differentiation in vitro, as assessed by path clamp experiments. On the other hand, MSCs derived from Wharton’s jelly and bone marrow were reported to display functional physiological properties only after in vivo differentiation upon implantation in situ.^45–47^ Jalali et al.^45^ showed that transplanting Wharton’s jelly-derived MSCs into the hippocampus of Parkinson’s disease rats, improved long-term potentiation (LTP) recordings from the hippocampal dentate gyrus areas. Yarar et al.^46^ showed that transplantation of BMSCs into a rat sciatic injury model enhanced recovery of sciatic nerve function, as assessed by electromyography and nerve conduction velocity testing. Hu et al.^47^ reported that BMSCs can be induced to differentiate into functional Schwann cells upon seeding on an electrospun aligned nanofiber scaffold, which in turn enhanced recovery of peripheral nerve injuries, as assessed by compound muscle action potential measurements upon nerve stimulation.

## Effects of direct electrical stimulation on the neurogenesis of mesenchymal stem/stromal cells

To date, there had only been few studies on the effects of direct electrical stimulation on the neurogenic differentiation of mesenchymal stem/stromal cells (MSCs). Thrivikraman et al.^48^ reported that the application of intermittent electrical stimuli enhanced the neural-lineage commitment of human MSCs on an electroconductive Polyaniline (PANI) substrate. Besides increased expression of neural markers such as βIII tubulin and nestin, there was also observed to be significant morphological changes in the form of filopodial elongation, after 7 days of electrically stimulated culture. In another study by Thrivikraman et al.^49^, it was demonstrated that application of a direct current electric field in the presence of electroactuated gold nanoparticles (GNPs), could also promote the neurogenic differentiation of human MSCs. The same study also identified G0/G1 cell cycle arrest, oxidative signaling, and elevated intracellular calcium ion levels as key upstream regulators of enhanced neurogenic differentiation promoted by direct current electrical stimulation in the presence of GNPs.^49^ Similar results were reported by Balikov et al.^50^, who observed upregulated expression of the neurogenic markers MAP2 and βIII tubulin upon electrical stimulation (cyclic voltammetry 0.5 V, 0.3 V, and 0.1 V, at 1 Hz, 3 Hz, and 5 Hz) of human MSCs on a graphene substrate. It was reported that the expression of βIII-tubulin was greatly enhanced by electrical stimulation in a voltage-dependent manner on unpatterned graphene, whereas on patterned substrates the expression of βIII-tubulin and MAP2 were significantly enhanced in all groups tested, versus the unpatterned substrate, but electrical stimulation did not further enhance the expression of these neural markers.^50^ Chudickova et al.^51^ reported that pulsatile electrical stimulation on a polycaprolactone (PCL) nanofibre scaffold led to enhanced neural differentiation of mouse adipose-derived mesencymal stem cells. More recently, in the study of Naskar et al.^52^, electric field stimulation was carried out on a coculture of murine myoblasts (C2C12) with human MSCs in a custom-designed polymethylmethacrylate (PMMA) based microfluidic device with in-built metal electrodes. Electrical stimulation resulted in human MSCs forming neurosphere-like clusters with elevated SOX2, nestin, and βIII-tubulin expression, and it was subsequently shown that intercellular calcium signaling played a key role in electrical field-induced neurogenesis. The study of Agarwal et al.^53^ reported enhanced neural differentiation of bone marrow-derived MSCs upon electrical stimulation (100 mV·mm^−1^) on a highly elastic, electroconductive, and immunomodulatory graphene cross-linked collagen cryogel designed for spinal cord regeneration. Additionally, the MSCs cultured on this scaffold under simulated inflammatory conditions in vitro exhibited high levels of immunosuppresive indoleamine 2,3 dioxygenase activity, which thus indicated its potential for neuroregeneration at inflammatory sites. Wu et al.^54^ developed a composite electrospun nanofiber scaffold by incorporating CNT into poly(p-dioxanone) (PPDO) nanofibers, which was demonstrated to accelerate human adipose MSC differentiation and maturation into Schwann cell-like cells, under a combination of electrical stimulation (50 mV/mm) and chemical induction. Besides upregulation of Schwann cell myelination-associated gene markers, there was also observed to be increased growth factor secretion.^54^ Zhou et al.^55^ enhanced neurogenic differentiation of human umbilical cord MSCs by electrical stimulation with direct current (100 mV·mm^−1^) on electrospun polypyrrole/polylactide composite nanofiber films, as manifested by upregulated expression of Nestin and NF-L. Similarly, Xu et al.^56^ enhanced neural differentiation of rat bone marrow-derived MSCs on conductive black-phosphorus-incorporated hydrogel by electrical stimulation (100 mV·cm^−1^).

## Enhancing neural differentiation of mesenchymal stem/stromal cells on electroactive scaffolds

Currently, there are also few studies on enhancing the neurogenesis of MSCs on electroactive scaffolds. The study of Guo et al.^57^ constructed a self-powered electrical stimulation-assisted neural differentiation system for MSCs by combining a poly(3,4-ethylenedioxythiophene) (PEDOT)-reduced graphene oxide (rGO) hybrid microfiber (80 μm in diameter) scaffold, together with a triboelectric nanogenerator (TENG) to supply pulsed electric simulation signals, which are triggered by human walking steps. MSCs cultured on the electroconductive rGO-PEDOT hybrid microfiber scaffold, not only exhibited improved neural differentiation potential, but also enhanced proliferative capacity. Similarly, Esmaeili et al.^58^ achieved enhanced neural differentiation of MSCs on a piezoelectric nanocomposite scaffold comprised of CoFe_2_O_4_ nanoparticles (CFO) incorporated within polyvinylidene difluoride (PVDF). Alizadeh et al.^59^ fabricated a soft electroactive hydrogel system composed of chitosan-oligoaniline, collagen and agarose, which promoted the differentiation of olfactory ecto-mesenchymal stem/stromal cells (OE-MSCs) into dopaminergic neuron-like cells. More recently, the study of Panda et al.^60^ fabricated piezoelectric nanocomposite scaffolds comprised of PVDF and multiwall-carbon nanotubes (MWCNTs), with or without BaTiO_3_ (BT) nanofillers. It was demonstrated that the PVDF/MWCNTs scaffold promoted differentiation towards the neuronal lineage, whereas the PVDF/BT/MWCNTs scaffold promoted differentiation towards the glial lineage, with Ca^2+^ oscillations, intracellular reactive oxygen species (ROS) and synaptic and gap junction proteins being identified to play key roles in directing lineage fate. It was hypothesized that the greater alignment and higher conductivity of the PVDF/MWCNTs scaffold is more conducive towards neuronal differentiation, whereas the moderate conductivity and high piezoelectricity of the PVDF/BT/MWCNTs scaffold is more conducive towards glial differentiation.^60^

## Carbon-based electroconductive scaffold materials—graphene and carbon nanotubes

To date, virtually all reported studies on electroconductive scaffolds for promoting neurogenesis of mesenchymal stem/stromal cells were based on carbon nanomaterials, in particular graphene and carbon nanotubes (cylindrical fullerenes). Although these are invariably composed of carbon atoms, the diverse shapes, sizes, variable surface chemistry and mechanical properties of these carbon allotrophic forms endow them with diverse properties,^61,62^ and are widely favored for tissue engineering applications, due to their mechanical strength, chemical stability, good biocompatibility, and high electrical conductivity. Moreover, their large surface area to volume ratio, and capacity to be functionalized with various chemical groups enables the loading and release of a diverse plethora of bioactive factors, including small chemical drugs, growth factors, nucleic acids, and proteins.^61,62^

Graphene is a 2D carbon allotrope that possesses a high level of mechanical flexibility, with an electrical conductivity within a magnitude of ≈10^3 ^S.cm^−1^.^63^ Depending on the specific requirements of the scaffold, graphene-based materials can be fabricated to be hydrophobic (i.e. reduced graphene oxide or fluorinated graphene), or moderately hydrophilic (i.e., graphene oxide). It must however be noted that hydrophobic graphene derivatives have more cytotoxic potential than hydrophilic derivatives, due to their ability to accumulate on cell membrane surfaces.

Cylindrical fullerenes, commonly referred to as carbon nanotubes, have also found wide application as scaffold materials for tissue engineering applications. These can be classified as single-walled carbon nanotubes (SWCNTs) comprising a single graphite sheet rolled into a tube with a diameter of one nanometer, or MWCNTs encompassing multiple graphene tubes surrounding the core of a SWCNT.^64^ MWCNTs are more often used, because the lower surface area of MWCNTs compared to SWCNTs, not only facilitates dispersal within a polymer matrix,^64^ but also results in lower cytotoxicity^64^ due to less cell membrane surface accumulation and subsequent internalization within the cytosol, which in turn reduces toxic interactions with cellular organelles, cytoplasmic components, and genomic DNA. Because the unique structure of carbon nanotubes allow encapsulation of other molecules, this has also prompted much interest in their use for drug delivery, in tandem with their application as tissue engineering scaffolds. Additionally, carbon nanotubes have also demonstrated anti-inflammatory properties,^65^ thus conferring additional advantages to their utility as tissue engineering scaffold materials. In the study of Bhardwaj and Saxena,^65^ it was reported that poly-dispersed acid-functionalized single-walled carbon nanotubes (AF-SWCNTs) were more readily internalized by activated T and B cells as compared to control resting cells, which suggests that AF-SWCNTs are naturally targeted to activated lymphocytes. Upon internalization, the AF-SWCNTs suppress T and B cell functions. However, the authors of this study admitted that it is still poorly understood how exactly AF-SWCNTs interacted with cytosolic molecules and internal cellular organelles to elicit the observed anti-inflammatory effects.

To date, a large number of studies within the scientific literature have demonstrated that electroconductive scaffolds based on carbon nanomaterials such as carbon nanotubes and graphene can enhance both neurogenesis in vitro, as well as neural tissue regeneration in vivo. Nevertheless, the overwhelming majority of such these studies were based on primary neurons, neural stem/progenitor cells and cell lines, with only a relatively small fraction of studies being focused on mesenchymal lineage cells, which will therefore be the focus of this review.

To date, there are only two known studies on utilizing electroconductive carbon nanomaterials for neural tissue engineering with dental and oral-derived mesenchymal stem cells. Simonovic et al.^66^ evaluated the effects of graphene dispersion (GD) and water-soluble single-walled carbon nanotubes (ws-SWCNT) on the neural differentiation of stem cells from apical papilla (SCAP), and found that both these carbon nanomaterials could upregulate the expression of key neural markers in SCAP. This study was however based on the soluble form of carbon nanomaterials, rather than their utility as a scaffold. In another study by Mansouri et al.^67^, it was demonstrated that the incorporation of graphene within alginate scaffolds enhanced the cell viability and adhesion of dental pulp stem cells (DPSCs), with minimal cytotoxic effects. Nevertheless, the neural differentiation of DPSCs was not evaluated, even though the scaffold was purported to be for neural tissue engineering applications.

## Enhanced neural differentiation of mesenchymal stem/stromal cells on graphene-based electroconductive scaffolds

### Graphene monolayers

Kim et al.^68^ described a unique graphene monolayer scaffold platform that provided a conducive microenvironment for the neural differentiation of human MSCs. Besides upregulation of neural markers and outgrowth of neurites, the MSCs were also observed to cluster together to form neurosphere-like structures on the graphene monolayer surface. Additionally, it was demonstrated that the MSCs derived neural-lineage cells on the graphene monolayer were sensitive to external ion stimulation, and that their neuronal properties were maintained even after detachment and re-seeding onto normal cell culture dishes, which thus indicated the enhanced maturity of the MSCs derived neuronal cells on the graphene monolayer. Similar results were reported by Lee et al.^69^, who observed that a smaller domain size of the graphene monolayer substrate increased hydrophilicity, which in turn improved cell-substrate adhesion, as well as enhanced neuronal differentiation of human MSCs.

### Graphene oxide and fluorinated graphene

Wang et al.^70^ reported enhanced neural differentiation of MSCs on fluorinated graphene, and also found that printed polydimethylsiloxane channel arrays on the fluorinated graphene substrate further enhanced the neurogenesis of MSCs, even in the absence of chemical inducers. Similarly, Kim et al.^71^ reported that certain combinatorial patterns of graphene oxide (GO) that mimicked interconnected/elongated neuronal networks, can promote the differentiation of human adipose-derived MSCs into the neural lineage. Additionally, graphene oxide substrate has also been reported to promote neurotrophic factor secretion and survival of human Schwann-like adipose-derived MSCs within in vitro culture, which in turn facilitated the ex vivo expansion of these cells for the treatment of peripheral nerve injuries.^72^

### Graphene and natural polymer composite scaffolds

Tasnim et al.^73^ reported that collagen-coated 3D graphene foams (GF) enhanced differentiation of mouse MSCs into dopaminergic neurons, as confirmed by upregulated neural marker expression, as well as increase in neurite length. Similarly, Guo et al.^74^ reported enhanced neurogenic differentiation of rat MSCs on a porous 3D composite scaffold composed of a reduced graphene oxide nanosheet layer assembled on porcine acellular dermal matrix (PADM), composed mainly of type I collagen.

### Graphene and synthetic polymer composite scaffolds

A number of electroconductive composite scaffolds comprising graphene and synthetic polymers for promoting neural differentiation of MSCs have been reported. Rawat et al.^75^ successfully differentiated MSCs into functional dopaminergic neurons using an electrospun polycaprolactone (PCL) and graphene nanocomposite scaffold, with the differentiated neurons exhibiting enhanced intracellular Ca^2+^ influx and dopamine secretion. Rasti Boroojeni et al.^76^ incorporated polyaniline graphene (PAG) within a hybrid PCL-gelatin nanofiber scaffold, which mimicked the native extracellular matrix and axon morphology. This provided a conducive microenvironment that enhanced differentiation of bone marrow MSCs derived neural stem cells to oligodendrocyte-like cells. Jakus et al.^77^ fabricated a 3D printable graphene composite scaffold consisting of majority graphene and minority polylactide-*co*-glycolide, and observed that even in the absence of neurogenic stimuli with simple growth medium, there was enhancement of human MSCs adhesion, viability, proliferation, and neurogenic differentiation, with significant upregulation of neural and glial gene markers. Moreover, human MSCs cultured on this scaffold exhibited highly elongated morphologies with features similar to axons and pre-synaptic terminals. Zhang et al.^78^ developed a novel cross-linked polyethylenimine (PEI) grafted graphene oxide hydrogel incorporated with SDF-1 chemokine, which accelerated both in vitro and in vivo neural differentiation of bone marrow MSCs. Similarly, the study of Qiao et al.^79^ also reported enhanced neural differentiation of bone marrow MSCs on an electroconductive graphene oxide-poly(acrylic acid) (GO-PAA) hydrogel.

### Graphene-augmented ceramic composite scaffolds

The only study to date was that of Kazantseva et al.^80^, which fabricated a composite graphene-augmented inorganic metal oxide ceramic scaffold. This highly anisotropic scaffold composed of ceramic nanofibres was able to induce spontaneous differentiation of human MSCs into the neural lineage without any specific differentiation media. Furthermore, this scaffold was also observed to suppress pro-inflammatory gene expression to some extent, whilst promoting monocyte taxis, which is particularly advantageous for facilitating neuroregeneration. Nevertheless, Kazantseva et al.^80^ admitted that their analysis of inflammatory factor expression by MSCs cultured on the scaffold did not indicate levels that might be relevant to severe immune reactions.

## Enhanced neural differentiation of mesenchymal stem/stromal cells on carbon nanotube-based electroconductive scaffolds

### Carbon nanotube scaffolds

Chen and Hsiue^81^ reported that carboxylated MWCNTs have low cytotoxicity and can promote neural differentiation of human bone marrow MSCs, in the absence of any exogenous differentiating factors, whilst suppressing the expression of osteogenic markers. Furthermore, upregulated neural growth factors secreted by the differentiating MSCs can also adsorb onto the carboxylated MWCNTs, thus trapping these factors to create a conducive microenvironment for long-term neural differentiation.^82^ The study of Park et al.^82^ demonstrated that linear network patterns on carbon nanotube-based films can further enhance the neural differentiation process by facilitating cell elongation and controlling the nuclear shape of human MSCs, leading to upregulated neural gene expression compared to bulk unpatterned carbon nanotube-based films. Another study by Kim et al.^83^ reported that nanoscale patterning on MWCNTs sheet resulted in a significant, synergistic enhancement of neural differentiation of human MSCs.

### Carbon nanotube composite scaffolds

A variety of different natural and synthetic materials have been utilized in the fabrication of carbon nanotube composite scaffolds. Lee et al.^84^ dispersed carbon nanotubes within collagen hydrogels to provide conducive 3D microenvironmental conditions for promoting neural differentiation of MSCs. Additionally, it was also observed that secreted neurotrophic factors, particularly brain-derived neurotrophic factor and nerve growth factor, were significantly upregulated by the incorporation of carbon nanotubes within the hydrogel. Ghorboni et al.^85^ coated multi-walled carbon nanotubes onto wet-electrospun poly-lactic acid 3D scaffolds that were already coated with the natural polymers alginate and gelatin. This composite scaffold enhanced the neural differentiation of human Wharton jelly-derived MSCs cultured in the presence of 1 mM valproic acid. Likewise, Mollania et al.^86^ also reported enhanced neural differentiation of MSCs on a carbon nanotube-pyrimethamine composite scaffold. Wang et al.^87^ fabricated an injectable, photoluminescent, carbon-nanotube-doped sericin hydrogel scaffold with programmable shape-memory property; which functionally promoted the neuronal differentiation of bone marrow MSCs. In the study of Shafiee et al.^88^, a hydrogel loaded with poly (lactic-*co*-glycolic acid) (PLGA) microspheres containing carbon nanotubes (CNT) and various biochemical differentiation factors was fabricated, as a biomimetic scaffold replicating the neural niche, to promote stem cell growth and differentiation. Not only did this biomimetic scaffold enhanced the proliferation of neural stem cells (NSCs) derived from MSCs, but also promoted the neuronal differentiation of these cells.

## Conclusions and future perspectives

Within the last decade, the design and fabrication of electroactive and electroconductive scaffolds for neural tissue engineering have progressed rapidly, facilitated by the development of new technology platforms such as 3D-printing,^89^ as well as the emergence of novel smart materials capable of responding to various physiological cues in vivo.^90^ Recently, there has even been a trend of the extra dimensionality of time being added to 3D scaffolds, resulting in 4D scaffolds with time-dependent variable properties.^91^

To date, it is unclear which type of neural lineages cells MSCs are prone to differentiate (with or without electrical stimulation), whether neurons, glia (oligodendrocytes), or astrocytes. This uncertainty and complexity are further compounded by the divergent MSC lineages from the various different tissue sources such as bone marrow, adipose tissue, wharton jelly, and oral/dental tissues. Probably, MSCs isolated from different tissue sources have different propensities towards different neural lineages, but to date, there has not yet been any rigorous or systematic study to address this. A major challenge is that characterization of neural-lineage markers is carried out on in vitro cultured MSCs on electroactive and electroconductive scaffolds, and that mature or terminal differentiation of the various neural lineages cannot take place in vitro. In many instances, the differentiating MSCs are arrested in vitro at the early progenitor stage that has the capacity to differentiate into either glia, astrocytes, or neurons. Moreover, it must be noted that MSCs from the different tissues sources are heterogeneous, with different sub-populations having propensities towards different neural lineages, which thus adds a further layer of complexity.

As previously mentioned, studies on neural differentiation of dental and oral-derived stem cells on electroactive and electroconductive scaffolds are very much limited to date. The only relevant data that can easily be extrapolated to these cells, come from the various aforementioned studies on the neurogenesis of bone marrow and adipose-derived MSCs on these scaffolds. Hence, much work still needs to be done to validate that the neural differentiation of dental and oral-derived stem cells can indeed be promoted by electroactive and electroconductive scaffolds. These would thus facilitate the clinical applications of these cells in neural tissue engineering and neuroregeneration therapy.
